# The Challenges of OSCC Diagnosis: Salivary Cytokines as Potential Biomarkers

**DOI:** 10.3390/jcm9092866

**Published:** 2020-09-04

**Authors:** Alexandra Roi, Ciprian Ioan Roi, Meda Lavinia Negruțiu, Mircea Riviș, Cosmin Sinescu, Laura-Cristina Rusu

**Affiliations:** 1 Department of Oral Pathology, “Victor Babeș” University of Medicine and Pharmacy Timisoara, Romania, Eftimie Murgu Sq. no.2, 300041 Timisoara, Romania; alexandra.moga@umft.ro (A.R.); laura.rusu@umft.ro (L.-C.R.); 2 Department of Anaesthesiology and Oral Surgery, “Victor Babeș” University of Medicine and Pharmacy Timisoara, Romania, Eftimie Murgu Sq. no.2, 300041 Timisoara, Romania; 3 Department of Propedeutics, “Victor Babeș” University of Medicine and Pharmacy Timisoara, Romania, Eftimie Murgu Sq. no.2, 300041 Timisoara, Romania; negrutiu.meda@umft.ro (M.L.N.); minosinescu@yahoo.com (C.S.)

**Keywords:** saliva, cytokines, biomolecules, biomarkers, oral cancer, early diagnosis, screening, noninvasive collection

## Abstract

Fast, economic, and noninvasive, molecular analysis of saliva has the potential to become a diagnostic tool of reference for several local and systemic diseases, oral cancer included. The diagnosis of Oral Squamous Cell Carcinoma (OSCC) can be performed using high specificity and sensibility biomarkers that can be encountered in the biological fluids. Recent advances in salivary proteomics have underlined the potential use of salivary biomarkers as early diagnosis screening tools for oral neoplasia. In this respect, over 100 salivary molecules have been described and proposed as oral cancer biomarkers, out of which cytokines are among the most promising. Besides being directly involved in inflammation and immune response, the role of salivary cytokines in tumor growth and progression linked them to the incidence of oral malignant lesions. This review summarizes the existing studies based on the use of salivary cytokines as potential oral cancer biomarkers, their involvement in the malignant process based on their type, and ther influence upon prognostic and metastatic rates.

## 1. Introduction

Diagnostics based on the analysis of saliva represent one of the most important promises of modern personalized medicine, with a potential impact on specific areas like screening, early diagnosis, therapy and post-therapy monitoring, and prognostic.

Salivary diagnosis has multiple advantages over traditional serum and tissue samples [1]. Besides being fully noninvasive and requiring little effort from the patient, analysis of salivary samples is a cost-effective approach, mainly due to easy harvesting, storage, and transfer. Saliva-based diagnostics has been the focus of present researches due to both proven good correlations of existing salivary molecules with blood levels and the existing connections with several systemic diseases, including oral cancer [1,2]. The permanent contact between saliva and the oral environment, especially malignant lesions, creates an opportunity for the development of screening, diagnostic, and monitoring tools with high sensitivity and specificity [3].

Oral cancer is a major global public health problem, ranking sixth among human malignancies, with a 5-year mortality rate, close to 50% [4]. Oral malignancies include all lesions encountered in the oral cavity, lip area, and pharynx, out of which 90% are squamous cell carcinomas (Oral Squamous Cell Carcinoma, OSCC) [5]. The 5-year survival rate of OSCC can be up to 90% in case of an early detection and proper treatment [5]. Oral cancer is often characterized by an insidious onset, difficult diagnosis, and fast evolution, frequently accompanied by metastasis and mutilating treatments. The high mortality and morbidity rate imply the need for an efficient screening method and the development of an early diagnostic tool [6]. Despite continuous advances in molecular biology and genetics, biopsy is still the ultimate, golden standard for oral cancer diagnostics [7].

Saliva is a complex mixture of major and minor salivary gland secretions, gingival crevicular fluid, serum, desquamated epithelial cells, bacteria and bacterial products, viruses and fungi, food debris, and other subcellular components, for which complexity is reflected by its biochemical and molecular composition [1,2]. Saliva contains water (95%), proteins (enzymes, antibodies, antimicrobial molecules, and cytokines), hormones, minerals, nucleic acids, and electrolytes [8,9]. The composition of saliva has the capacity to reflect tumor characteristics through a variety of specific biomarkers [10]. Several studies have shown that the changes determined by OSCC may be detected in saliva at the genomic, transcriptomic, and proteomic levels [11]. Also, recent proteomic studies of saliva provided new information that correlates salivary proteins/peptides in general and cytokines in particular with various pathological states [12,13,14,15,16]. The study performed by Kosaka et al. [14] mentions a strong relationship between the increased interleukin (IL)-6 and tumor necrosis factor (TNF)-α levels and the incidence of atherosclerosis found in their study group [14]. Increased salivary cytokine levels were also reported by Carvalho et al. [15] in patients diagnosed with oral lichen planus in comparison to the control group. Koizumi et al. [16] focused on analysis of a panel of salivary cytokines in non-small cell lung cancer patients, and their results showed increased levels of salivary interleukin-6 (IL-6), interleukin-8 (IL-8), interleukin-10 (IL-10), interleukin-7 (IL-7), interleukin-1b (IL-1b), tumor necrosis factor (TNF), platelet-derived growth factor-BB, C-X-C motif chemokine ligand 10 (CXCL10), C-C motif chemokine ligand 3, and C-C motif chemokine ligand 4 compared to the healthy control group.

A biomarker can be linked to the presence of a certain disease, and it is associated with existing biochemical, genetic, or cellular alterations. The identification of such biomarkers in saliva can influence fast diagnosis, progression, prognostic, and the posttreatment monitoring of OSCC by offering an alternative to the standard procedure [1]. An ideal biomarker should provide high sensitivity and specificity, allowing accurate diagnosis. Although a wide array of biomarkers has been investigated, cytokines represent a target due to their intercellular communication role. An abnormal expression of different cytokines can be a response to a pathological process. The small proteins called cytokines are responsible for local cell interactions and have an influence upon the cells that secreted them as well as upon other cells located further or nearby. Different cytokines can develop similar functions, as they can influence the target cell to produce even more cytokines [17,18].

Current evaluation of the salivary proteome promises a new method for the diagnosis, evolution monitoring, and prognostic of several diseases, among which is primary Sjögren’s syndrome [19]. The salivary proteome represents a collection of 1166 molecules encountered in the saliva from submandibular, sublingual, and parotid glands [20]. Studies focused on the salivary cytokine levels in patients diagnosed with primary Sjögren’s syndrome and the study performed by Ohyama et al. [21] reported a significantly higher concentration of Th1 cytokines (IL-2, IL-6, IL-8, TNF, IFN- *γ*, and IL-1α) and Th2 cytokines (IL-10, IL-5, and IL-4) in the saliva of the included patients diagnosed with Sjögren’s syndrome. Another focus was upon the existing association of different salivary cytokine levels and the severity of cystic fibrosis airway disease. Cataldo et al. [13] outlined in their results that an increase of salivary IL-8, IL-6, and TNF-α was identified in the cystic fibrosis group compared to the controls.

Despite the fact that most of the saliva’s analyte concentration can be lower compared to the blood samples, in the case of diagnosis of OSCC, this aspect is not considered a limitation as the local oral lesion is responsible for the specific changes in the saliva composition [22]. Still, the salivary liquid biopsy lacks important information related to the exact concentration of specific biomarkers that can certify a positive diagnosis. An important aspect related to the saliva samples and their use for diagnosis is the collection method, time, and type of saliva (stimulated or unstimulated saliva). The reason why the saliva sampling must be standardized is the fact that more factors can influence its composition, such as sex, age, oral hygiene, and diet [23]. Circadian variations also have an impact upon the secretion of saliva; this is the reason why it is suggested that the sampling time should be performed between 8 AM and 10 AM, after 12 h from the last meal [24]. Although there are two types of saliva, gland-specific and whole saliva, for OSCC diagnosis, the whole stimulated saliva is more accurate as the concentration of analytes is more representative [24]. For correct biomarker quantification, the time of the sampling analysis should be under 5 min; otherwise, a degradation process can occur [22].

The development of a particular diagnostic approach based on the correlation of salivary cytokine levels and the incidence of OSCC was suggested by several studies as a screening option in order to improve early detection [25,26]. The aim of this review is to critically analyze the existent data related to the implication of salivary cytokines as potential biomarkers for OSCC and to outline the importance of their assessment as an early diagnostic tool. Previous studies have discussed the direct involvement of altered cytokine levels, both in serum and in saliva, encountered in OSCC patients [27,28]. The potential of cytokines has been studied, and their involvement in carcinogenesis directly associated with the host response to malignant proliferation, disease progression, and mortality [29,30]. The fact that these cytokines have a role in multiple actions (cell proliferation, angiogenesis, tissue remodeling, and differentiation) and are also responsible for the apoptosis process suggest that the occurrence of an abnormal alteration could lead to modified actions that sustain the malignant process [31,32].

### 1.1. Cytokines Biology

One of the main roles of cytokines is to mediate and influence the immune system [33]. Their role in the mediation process of oral local inflammations even in a low concentration is an important characteristic [34]. Locally, in the oral environment, the epithelial cells of the mucosa continuously produce cytokines as a response to various oral infections [35,36]. An elevated cytokine level as a response to multiple microorganisms can determine the appearance of a chronic inflammation that can secondarily lead to oral cancer [37].

Cytokines are intercellular signaling proteins that are responsible for cell proliferation, growth regulation, angiogenesis, and tissue repair [38]. Cytokines can be broadly classified into two groups: pro-inflammatory (IL-1b, IL-6, IL-8, granulocyte-macrophage colony stimulating factor (GM-CSF), tumor necrosis factor alpha (TNF-a), and transforming growth factor beta (TGF-*β*)) and anti-inflammatory (IL-2, IL-12, IL-4, IL-10, and interferon gamma (IFN-*γ*)) [18].

The antifibrotic effects of several cytokines were evaluated in an in vitro study by Chen et al. [39] as vocal fold scar treatment. Their results showed that IL-6 and IL-10 displayed an antifibrotic and anti-inflammatory action in vitro, providing a possible new solution in cases of vocal fold scarring.

### 1.2. Cytokines in OSCC

OSCC has a complex etiopathogenesis and involves the existence of a certain lifestyle and specific risk factors (tobacco, alcohol, and Human Papilloma Virus) [4]. A common characteristic of all malignancies, with no exception from OSCC, is the implication of two decisive processes: an abnormal differentiation of the malignant cells and an inflammatory response [10]. Several studies showed that the ones implicated in mediation of the inflammatory process are the cytokines with a key role in tumor progression [17].

Two groups of cytokines (pro-inflammatory and anti-inflammatory) are secreted in the local tumor environment by the immune and malignant cells [18]. Their actions coordinate the immune tumor response and have an influence upon the proliferation and growth (pro-inflammatory cytokines) and an anti-suppressive action against malignant cells (anti-inflammatory cytokines) [18]. The results of existing studies linked the pro-inflammatory and the pro-angiogenic cytokines IL-8, IL-6, TNF-β, and IL-1β with an aggressive behavior of OSCC [40]. As they promote tumor growth, a higher concentration of these cytokines is associated with a higher metastatic risk as well as the development of larger tumors [32].

### 1.3. Available Technologies for the Identification of Salivary Biomarkers

The most used immune-based assays in published studies for the evaluation of salivary samples are enzyme-linked immunosorbent assay (ELISA) and bead-based suspension array (Bio-Plex 200 system, Bio-Rad Laboratories, Inc., Hercules, CA, USA) [41]. The advancements made and developments in technology managed to successfully relate the levels of several salivary biomarkers with the diagnosis of OSCC. Enzyme-linked immunosorbent assay (ELISA) is a specific method that targets specific molecular parts and detects arrays from different samples. It can be either a direct, indirect, sandwich, or competitive ELISA method, depending on the procedure of antibody detection. An advantage of the ELISA technique is the fact that it can be adjusted for an increased sensitivity and specificity.

Multiplex assays are based on the property of detecting targeted analytes in biological samples. The type of samples can be saliva, serum, or supernatants collected from in vivo or in vitro cell lines. Several multiplex systems are used for the diagnosis of cancer, cardiac diseases, allergic and autoimmune diseases, and infectious diseases. They can be used in order to identify cell signaling and antibodies and to perform cytokines and chemokine analysis [41]. One of the multiplex systems is the Luminex technology, and it is based on fluorescent beads that can be coated with specific reagents in order to perform different assays. Multiplex cytokines assays have a series of advantages compared to traditional tests, such as an increased sensitivity and an enhanced quantification range, allowing a reduced number of samples by performing multiple protein arrays in a single sample, reducing costs and time [42].

## 2. Material and Methods

The search for the required articles for this review was performed using PubMed, Web of Science, Medline, Google Scholar, Scopus, and EMBASE. Our review included the results of published research articles between years 2005 and 2019. The search terms used were “human saliva”, “salivary proteins”, “cytokines”, “oral cancer”, “oral cancer screening”, “head and neck cancer diagnosis”, “proteomics”, “oral cancer diagnosis”, “noninvasive oral cancer screening”, “biomarkers”, “oral cancer biomarkers”, and “salivary cytokines”, all of them in various combinations (“oral cancer biomarkers”, “salivary cytokines as diagnostic tools”, “salivary cytokines as biomarkers”, and “salivary proteomics”). The articles included in this review were human-based salivary studies based on OSCC study groups and controls. Case reports, letters to editors, opinions, and author responses were excluded.

## 3. Results

### 3.1. Cytokine Biomarkers in Saliva of Patients with OSCC

Many studies performed in the past decade focused on the identification of cytokine concentration in the saliva of OSCC patients, using the ELISA method as an adjuvant in the detection of potential protein biomarkers [43]. The results of Tang et al. [44] stated that an abnormal cytokine level can play an important role as a biomarker for OSCC. Altered cytokine levels have multiple functions besides those associated with immune response or inflammation. The results reported that they are also involved in cell growth, tumor progression, and the aggressive character of the tumor [45].

The study conducted by Lee et al. [9], based on two groups of patients, the OSCC group and the non-cancer group, showed that the levels of eight biomarkers (plasma IP-10 and salivary eotaxin, IFNg, macrophage inflammatory protein-1b (MIP-1b), IL-1b, IL-6, IL-8, and TNF-a) were significantly higher in the OSCC group that in the control one. Also, when the level of salivary biomarkers from the OSCC in stage I/II was compared to a group with stage III/IV OSCC patients and with a healthy control group, the results revealed that there were significant differences in the levels of salivary IFN-g, growth regulated oncogene (GRO), IL-1b, IL-6, IL-8, MIP-1b, and TNF-a. Except the level of IFN-g, the other six biomarkers showed significant differences between stage I/II OSCC patients and controls [9].

### 3.2. Role of IL-6 and IL-8 Salivary Levels in OSCC Patients

Research indicates that IL-6 concentrations in the serum and saliva of patients with oral neoplasia and premalignant lesions are significantly higher in comparison with the control group [40,46]. The report of the study performed by Rhodus et al. [47] shows that patients diagnosed with OSCC and oral premalignant lesions had higher salivary IL-6 levels than the control group [47].

A study performed by Sato et al. [48] regarding changes that occur in the saliva of OSCC patients and the salivary IL-6 level during the OSCC treatment phase aimed to perform an accurate correlation with the clinical factors. In this study, twenty-nine patients diagnosed with OSCC were enrolled. Their stimulated saliva was collected just after hospitalization (period 1), before the main treatment (period 2), and at the time of discharge (period 3) and was compared to a control group. Measuring IL-6 concentrations with the help of a highly chemiluminescent enzyme immunoassay proved that the IL-6 levels in the stimulated saliva of OSCC patients was significantly higher than in the control group and that it was not reduced at discharge [49]. These results suggest that the release of IL-6 into the saliva of OSCC patients is higher and that it can influence maintenance of the disease. Proinflammatory cytokines are directly involved in the carcinogenesis process, and one of the characteristics of IL-6 is that it inactivates the p53 tumor suppressor gene [50,51,52]. It has been suggested that tumor size (T-stage) is an independent predictor of survival and that large tumors may secrete higher amounts of IL-6 [32]. Santo et al. [48] in their study have not found significant correlations between IL-6 concentrations and the tumor size.

Hamad et al. [51] in their study reported that the salivary and serum levels of IL-8 of the included OSCC patients were significant higher compared to the controls and proved to be a useful tool for diagnosis. The levels of IL-6 were found to be also higher in the serum samples, while in the salivary ones, a significant difference was also notable compared to healthy subjects. Another study performed by Saheb et al. [53] compared the levels of TNF, IL-8, and IL-6 between the group of patients with OSCC and the controls based on age and sex. Their study concluded that only IL-6 levels were statistically significant, and that other biomarkers, although their levels were raised compared to the control group, had no statistically significant differences [51] (Table 1). Val et al. [54] in their study aimed to quantify the variation of multiple cytokines in patients diagnosed with oral squamous cell carcinoma. Conducting a longitudinal-prospective study with a case-crossover design, non-stimulated saliva samples were analyzed and assessment of the cytokines was performed. The results showed that the presence of oral squamous cell carcinoma influenced the salivary levels of IL-8, IL-6, VEGF, and IL-1β, determining an increased concentration. The largest prospective controlled study was conducted by Arduino et al. [55], focusing on the correlation between the serum and salivary levels of IL-6 and IL-8 before any treatment and on further monitorization of the survival rate and recurrence of OSCC. Csosz et al. [56] targeted 14 proteins that were reported to be associated with the saliva of OSCC patients. Their results confirmed an increased level of salivary and serum cytokines IL-6 and IL-8 compared to the matched controls. Another study that assessed the high salivary concentrations of IL-8 in OSCC patients compared to a healthy control group was conducted by Glebber et al. [57]. Other results were obtained by Kaur et al. [58] when they evaluated the levels of these particular cytokines in salivary samples from patients with premalignant lesions, showing the fact that IL-8 had significant differences with higher levels in comparison to the healthy group. Other research performed on patients diagnosed with oral leukoplakia showed that the levels of IL-6 and TNF in the saliva can be used as a proven significant clinical biomarker [40].

A study that focused on patients diagnosed with OSCC in T1/T2 stage revealed that IL-8 was elevated in the salivary samples, as was IL-6 in the serum ones. As a diagnosis option based on these cytokines, the association of an elevated IL-8 salivary level with the serum IL-6 elevated levels showed a very high accuracy [59].

Targeting salivary IL-6 and the changes in the concentration associated with OSCC patients, Paneer et al. [60] concluded that high levels of IL-6 were related to OSCC patients, suggesting that increased local production by the tumor cells can occur.

### 3.3. Role of IL-10 and IL-13 Salivary Levels in OSCC Patients

As potential prognostic biomarkers, IL-10 was of particular interest as its function is involved in the differentiation of T cells. Tumoral invasion is possible through the contribution of IL-10 by inhibiting the function of macrophages and dendritic cells that are responsible for presenting the tumor antigen to T lymphocytes [65]. Also, the high levels of IL-13 were associates with several tumor types such as head and neck carcinoma, Kaposi’s sarcoma, renal carcinoma, and ovarian cancer [66].

In a cross-sectional study performed by S. Aziz et al. [67], 30 diagnosed and untreated patients (histopathological confirmed) with OSCC were included. These patients were split in three groups based on the histopathological cell differentiation (grade 1, grade 2, and grade 3), and the results were compared to those from a control group. Unstimulated saliva was collected from all groups and underwent a multiplex immunoassay in order to quantify the salivary levels of IL-13, IL-1RA, IL-4, and IL-10. The purpose of the research was to compare the salivary immunosuppressive cytokine levels, and it was observed that between the OSCC group and the control group was a statistically significant difference regarding the involvement of IL-13 levels and IL-10 levels (Table 2). Also, further results showed that the levels of IL-1RA found in the saliva showed a high level among the patients having poorly differentiated (grade 3) OSCC tumors, with a statistically significant difference. IL-10 was found to be present in significantly higher levels in patients with well-differentiated tumors in comparison to the control group. This study indicates that IL-1RA, IL-13, IL-4, and IL-10 had significantly higher levels in patients with OSCC. The levels of cytokines IL-13 and IL-10 were found to have elevated salivary levels in OSCC patients, suggesting a strong potential that could transform them in diagnostic and prognostic biomarkers [68,69]. Goncalves et al. [70] conducted a study that evaluated IL-10 as a potential immunosuppressive mediator in OSCC patients compared to healthy controls. Their results detected a high level of IL-10 in the saliva and tumor environment, distinguishing the OSCC patients from the controls. However, a study by Kaskas et al. [71] showed different results that concluded that IL-13 had lower serum levels in head and neck cell carcinoma in comparison to the control group.

## 4. Discussion

The epidemiology of oral squamous cell carcinoma outlines that the 5-year survival rate of patients diagnosed in stage I is 72–90%, in stage II is 39–85%, in stage III is 27–70%, and in stage IV is 12–50% [70,75]. These statistics imply the necessity to develop better approaches for early diagnosis and better prediction of the aggressiveness of tumors.

The use of biomarkers identified in both serum and saliva in order to diagnose OSCC has been widely embraced by researchers. However, limitations do exist as the correlations between the two body fluids are not possible and the associated systemic pathologies can have an important impact. This present review focuses on several important salivary cytokines proven in the involvement in pathogenesis and diagnosis of OSCC, and by choosing IL-6, IL-8, IL-10, and IL-13 as the subject of many research papers, we discuss how these types of cytokines influence the malignant process through their main yet different characteristics.

Studies have outlined that IL-6, IL-8, VEGF, IL-1b, and TNF-α are involved in the initial process of OSCC and that the present antibodies against IL-6 and p53 can be potential salivary biomarkers [76]. Although, there have been more than 100 salivary biomarkers identified, the need for a standardization method brings to light the different outcomes of research studies due to the lack of a collection and storage protocol [77].

Using saliva as a potential diagnostic biofluid has been accepted, and its many advantages over other specimens like blood, exfoliated cells, and urine are uncontestable. The importance of salivary biomarkers represents the key for a future diagnostic approach. The detection of cytokines in the saliva of OSCC patients brings to light the fact that they contribute through their pro-inflammatory functions to the initiation and evolution of oral cancer. The biological and molecular factors have been studied due to their prognostic characteristics [78]. The cytokine’s influence in the inflammatory and immune response of oral mucosa cannot be underestimated, playing an important role in disease progression. IL-6 is a cytokine with multiple functions that is synthesized as a response to various stimuli by different cells [79]. A complex bond exists between the proinflammatory and anti-inflammatory cytokines that exist in the tumor environment and that are being secreted not only by the immune cells but also by certain tumor cells [18]. Previous literature suggests that anti-inflammatory cytokines are immunosuppressive agents that counteract the proliferative potential and modify antitumor immune responses [62]. Besides their immunosuppressive main role, these cytokines mediate the process of differentiation from TH1 cells to TH2 cells, mainly through downregulation of antitumor immunity [63]. The immunosuppressive cytokines such as IL-4 and IL-10 have been long investigated in cervical carcinoma [64], while the IL-1 receptor antagonist has been investigated in relation to epilepsy [67]. Although their immunomodulatory role in preventing tumor rejection sustaining tumor growth is a concern, the lack of more information regarding these potential biomarkers needs to be studied intensively in association with OSCC.

Analyzing the precise involvement of these specific cytokines can lead to advancements and new therapeutic strategies for early diagnosis and prognostic of this disease. As the results of Lee et al. [9] stated, alterations of the levels of various potential cytokine biomarkers in the microenvironment surrounding oral malignant lesions may not be as strongly relevant in systemic blood samples. Taking into consideration these results, the importance to standardize biomarkers in saliva becomes a priority.

Different biomarkers can be directly associated with the malignant process, or they can be the response of the human body towards a malignant proliferation [70]. The studies analyzed in this review focused on the evaluation of cytokine levels of IL-6, IL-8, IL-10, and IL-13 and their potential role as OSCC biomarkers. All included studies were clinical ones, and the main purposes were to quantify the potential different levels of these cytokines in direct relation with OSCC groups of patients.

Oral cancer screening is an important step in order to improve early diagnosis, and the use of salivary biomarkers promises to open a new perspective in order to accomplish this.

The studies that targeted salivary cytokines as OSCC biomarkers reported a strong relationship between their high levels and the presence of OSCC [18,49,51,53,54,55,56,57,60,61,62,63,64]. Further challenges consist in ruling out other possible causes that could determine the increase of salivary cytokines, such as periodontitis, cystic fibrosis, Sjögren’s syndrome, and acute stress [13,19,71,72]. Taking these facts into consideration, besides an oral cancer screening tool that utilizes cytokine biomarkers, it is important to evaluate general health and oral statuses. Nevertheless, the salivary cytokine biomarkers would also play an important role in disease monitoring and recurrence of OSCC. Russo et al. [80] stated that posttreatment monitoring based on the levels of salivary cytokines is dependent on the type of treatment that the patient underwent, as radiation can influence cytokine levels by increasing them.

IL-6 is a proinflammatory cytokine that is actively involved in the growth of various types of cancer, influencing and increasing the rate of metastasis [81,82]. More studies have revealed that IL-6 can have two effects at the same time: on one side, it can stimulate the growth of certain cells, while on the other side, it can inhibit growth of other cell types [48]. The characteristics of this cytokine is that it can inhibit or stimulate different types of tumors [40].

IL-8 is a type of cytokine that has chemotactic activity for neutrophils, basophils, eosinophils, monocytes, mast cells, dendritic cells, lymphocytes, and natural killer (NK) cells [9]. Until now, multiple studies have been reported in head and neck cancer with different results, contradicting conclusions and inadequate follow-up in different tumor types [59,78].

IL-8 is released by neutrophils and macrophages as a response to multiple external and internal agents, including environmental, chemical, and different stress factors, performing a pro-inflammatory action. Once IL-8 is activated, it can influence the activity of the receptor CRCX-1 and the receptor CRCX-2. These types of receptors are found on macrophages and neutrophils that are tumor-associated, suggesting that IL-8 levels can be an important chemokine in relation with cancer cells [32,83]. The cancer process is characterized by neutrophil recruitment, proliferation, angiogenic potential, the movement of vascular endothelial cells, and protection from metastasis and apoptosis [84]. The involvement of IL-8 in cancer pathogenesis can be justified by the fact that the treatment of disease with chemotherapeutic agents reduces the expression of this type of cytokine [18,49]. Also, it has been discussed that IL-8, besides the direct implication in tumor proliferation, has a significant action upon transition of the epithelium [85].

The studies focusing on the potential of the cytokine IL-6 and IL-8 as salivary biomarkers and their modified concentrations outline higher salivary and serum levels encountered in the OSCC groups compared to the control ones [46,50,54,55,56,57,65,67,86]. The higher levels of cytokines identified in the samples of OSCC patients proved that they cannot be associated with any other causes that could modify the levels, as IL-8 levels may differ with ethnic and genetic differences or lifestyle [83]. The cytokine levels can also be modified by the existence of periodontitis or gingivitis, but none of these mentioned diseases can influence such elevated levels as those seen associated with the OSCC groups. A study performed by Punyani et al. [62] related to the pro-angiogenic and inflammatory characteristics of IL-8 showed that, within the 25 samples obtain from OSCC patients, the elevated levels of IL-8 confirmed the role in angiogenesis and progression of this type of cytokine [53]. In another study, Saheb et al. [53] compared the salivary levels of IL-8 and IL-6 between patients diagnosed with OSCC, with controls sex- and age-matched. Their results showed that only the IL-6 levels were statistically elevated; IL-8, although having increased levels, showed no statistical difference [53]. Kaur et al. [66] identified elevated levels in the salivary and serum samples of IL-6, IL-8, and TNF in patients diagnosed with oral leukoplakia, oral submucous fibrosis, and lichen planus, suggesting the high potential of these cytokines as biomarkers as they can be directly associated with oral precancerous lesions. The results of the meta-analysis performed by Shree et al. [87] presented the fact that IL-8 is defined as a biomarker with a moderate sensitivity and specificity and acts as an angiogenic factor in tumors, while IL-6 influences the tumor development.

In the cancer pathogenesis, IL-10 was also proven to be involved in anti-inflammatory and immunosuppression functions. Multiple studies associated the elevated levels of IL-10 produced by the tumor cells with different types of malignancies and aggressive behavior that should influence the surgical management [88,89]. In the study conducted by Ali et al. [90], IL-10 levels were found increased in the OSCC samples compared to the control group. Also, these results were similar to those reported by Fujieda et al. [91] and Hamzavi et al. [74]. However, Chandler et al. [92] found a lower occurrence of IL-10 that can be related to the differences in the binding to antigen processes. Elevated serum levels of IL-10 were related to advanced stages of OSCC [90,93]. In the study performed by Nelson et al. [94], the salivary levels of IL-10 in relation with cervical cancer were evaluated, and they concluded that there were no significant differences between the cancer group and the controls, suggesting that IL-10 might not be a proper biomarker.

In the study conducted by Aziz et al. [67], the high salivary levels of IL-10, IL-13, and IL-4 were found in the OSCC group. Oppositely, the results reported by Kaskas et al. [73] showed lower levels of IL-13 in head and neck squamous cell carcinoma compared to the healthy control group.

The responses of the immune system in OSCC patients are deficient and favor the development and progression of malignancy [95,96,97]. As OSCC is in a direct relationship with chronic inflammation, it has been proven that the cytokine levels are altered [98,99], providing a proper environment for the proliferation and growth of malignant cells, mainly based on the alteration of the immune-surveillance program [61].

This present review outlines the importance of an early diagnosis and the necessity to integrate a noninvasive approach in order to improve the prognostic. The current advancements made in proteomic studies have the potential to overcome the existent milestone and to extend the era of cancer diagnosis by including the information that salivary cytokines have to offer.

## Figures and Tables

**Table 1:** Summary of included studies related to interleukin (IL)-6 and IL-8 and Oral Squamous Cell Carcinoma (OSCC).

Cytokine Biomarker	Matrix	Validation Methods	Biological Sample	Results	*p* Value	Reference
IL-8	Saliva	ELISA	Control (n = 13) OSCC (n = 13)	Salivary IL-8 levels were significantly elevated in OSCC patients compared to the control group.	<0.001	[18]
IL-6	Saliva	ELISA	Control (n = 13) OSCC (n = 13)	Salivary IL-6 levels were significantly higher compared to the control group.	<0.001	[18]
IL-6	Saliva	ELISA	Control (n = 20) OSCC (n = 19)	Elevated levels of IL-6 were identified in the OSCC group.	<0.05	[49]
IL-8	Saliva	ELISA	Control (n = 20) OSCC (n = 19)	High level of IL-8 were identified in the OSCC group.	<0.05	[49]
IL-6	Saliva Serum	ELISA	Control (n = 20) OSCC (n = 30)	Significant difference was encountered between the salivary and serum IL-6 levels in the OSCC group and the control one.	<0.001	[51]
IL-8	Saliva Serum	ELISA	Control (n = 20) OSCC (n = 30)	Highly significant difference was noted in the IL-8 salivary and serum levels of the OSCC group compared to the control one.	<0.001	[51]
IL-6	Saliva	ELISA	Control (n = 30) OSCC (n = 30)	Increased salivary levels of IL-6 were identified in the OSCC group compared to the control one.	<0.001	[53]
IL-8	Saliva	ELISA	Control (n = 30) OSCC (n = 30)	Highly significant difference was identified in the IL-8 levels compared to the control one.	<0.0001	[53]
IL-6	Saliva	Bio-Plex multiplex	Control (n = 21) OSCC (n = 20)	IL-6 concentration was higher in the OSCC group compared to the control (T0).	0.005	[54]
IL-8	Saliva	Bio-Plex multiplex	Control (n = 21) OSCC (n = 20)	IL-8 concentration was higher in the OSCC group compared to the control one (T0).	0.004	[54]
IL-6	Saliva Serum	ELISA	Control (n = 52) OSCC (n = 52)	Higher levels of IL-6 before the treatment were associated with the survival rate; higher levels were identified in patients with OSCC compared to the controls.	<0.001	[55]
IL-8	Saliva Serum	ELISA	Control (n = 52) OSCC (n = 52)	IL-8 was identified in higher concentrations in the saliva and serum of patients with OSCC.	0.010	[55]
IL-6	Saliva Serum	Luminex assay ELISA	Control (n = 9) OSCC (n = 26)	The IL-6 level was significant higher compared to the age-matched controls.	0.0002	[56]
IL-8	Saliva	Luminex assay ELISA	Control (n = 9) OSCC (n = 26)	IL-8 had a slightly higher concentration in the OSCC group compared to age-matched controls.	0.1087	[56]
IL-8	Saliva	ELISA	Control (n = 60) OSCC (n = 60)	The concentration of IL-8 was significantly higher in the OSCC group compared to the control.	<0.0001	[57]
IL-6	Saliva	ELISA	Control (n = 25); OSCC (N = 25)	Salivary IL-6 is significantly elevated in OSCC compared to the healthy control group.	<0.001	[60]
IL-6	Saliva Serum	ELISA	Control (n = 100) OSCC (n = 100)	Salivary and serum IL-6 are significantly elevated in OSCC compared with healthy control group.	<0.05	[61]
IL-8	Saliva	ELISA	Control (n = 25); OSCC (n = 25)	Salivary IL-8 is significantly elevated in the OSCC group compared with the healthy control group.	<0.0001	[62]
IL-8	Saliva Serum	ELISA	Control (n = 100) OSCC (n = 100)	Salivary IL-8 is significantly elevated in the OSCC group, and serum IL-8 is also significantly elevated in OSCC compared with controls.	<0.05	[63]
IL-8	Saliva	Luminex ELISA	Control (n = 20) OSCC (n = 20) Control (n = 42) OSCC (n = 40)	IL-8 may be used as individual markers of OSCC. IL-8 was expressed at significantly higher levels in OSCC subjects than in the healthy controls.	<0.098	[64]

**Table 2:** Summary of included studies related to IL-10 and IL-13 and OSCC.

Cytokine Biomarker	Matrix	Validation Methods	Biological Sample	Results	*p* Value	Reference
IL-10	Saliva	MILLIPLEX (4-plex) Human Cytokine/Chemokine Assay kit	Control (n = 33) OSCC (n = 30)	IL-10 levels in OSCC individuals were found to be higher than the healthy counterparts and could be utilized as diagnostic and prognostic markers in OSCC patients.	0.004	[67]
IL-13	Saliva	MILLIPLEX (4-plex) Human Cytokine/Chemokine Assay kit	Control (n = 33) OSCC (n = 30)	IL-13 levels were elevated in the OSCC group in comparison to the healthy one.	0.010	[67]
IL-10	Saliva Serum Tissue	ELISA IHC	Control (n = 24) OSCC (n = 30)	IL-10 tissue expression was statistically significant higher in the OSCC group, but no statistical differences were observed in serum or salivary levels.	0.001	[72]
IL-10	Saliva Serum Tissue	ELISA IHC	Control (n = 5) OSCC (n = 20)	Il-10 levels in serum had no significant difference in the OSCC and the controls; OSCC biopsies indicated immunoreactivity to IL-10, while normal samples were immune-negative.	<0.05	[73]
IL-10	Saliva Serum	ELISA	Control (n = 40) OSCC (n = 78)	IL-10 level was higher in the OSCC group than in controls in the salivary and serum samples.	<0.05	[74]
